# Phase Separation in Regulation of Autophagy

**DOI:** 10.3389/fcell.2022.910640

**Published:** 2022-05-02

**Authors:** Yi Lu, Chunmei Chang

**Affiliations:** ^1^ Tongji University Cancer Center, Shanghai Tenth People’s Hospital, School of Medicine, Tongji University, Shanghai, China; ^2^ Institute of Metabolism and Integrative Biology, Fudan University, Shanghai, China

**Keywords:** autophagy, phase separation, selective autophagy, autophagosome formation, autophagy substrate, cargo receptor

## Introduction

Macroautophagy (hereafter referred as autophagy) is a highly conserved degradation pathway by which the cytoplasmic materials are sequestered by the double-membrane vesicles named autophagosomes, and delivered to lysosomes for degradation and recycling (Nakatogawa et al., 2009). Autophagy is initiated by the *de novo* formation of a double membrane phagophore (also known as isolation membrane) around intracellular substrates, the phagophore grows into an intact autophagosome, and the autophagosome fuses with lysosome. Autophagy is tightly controlled by diverse signaling molecules. Dysfunction of autophagy is often linked to a variety of diseases, including neurodegenerative diseases, cancer, metabolic disorders, and inflammation (Levine and Kroemer, 2019).

The hallmark of autophagy is autophagosome formation, which involves nucleation, expansion, and closure of the phagophore (Melia et al., 2020; Nakatogawa, 2020). A number of autophagy related proteins (ATG) cooperate to mediate autophagosome biogenesis. In mammalian cells, autophagosome nucleation is typically driven by the ULK1 (unc-51-like kinase 1) complex, the counterpart of the Atg1 complex in yeast. The class III phosphatidylinositol 3-kinase complex I (PI3KC3-C1) is activated to generate phosphatidylinositol-3-phosphate (PI(3)P), which recruits the downstream effector WIPIs (WD-repeat protein interacting with phosphoinositides). WIPIs in turn recruit and activate the conjugation machinery to mediate the lipidation of ATG8 family proteins. The lipid transporter ATG2, the scramblase ATG9 and lipidated ATG8 proteins contribute to phagophore expansion, and the ESCRT machineries are recruited to finalize the closure to form an intact autophagosome (Mizushima et al., 2011; Hurley and Young, 2017; Chang et al., 2021a).

Recent studies show that liquid-liquid phase separation plays important roles in different steps of autophagy. Phase separation is a process in which biomacromolecules such as proteins and nucleic acids can coacervate into liquid-like membrane-less condensates, which is driven by weak multivalent interactions between modular interaction domains or intrinsically disordered regions (IDRs) containing low complexity amino acid sequences. Phase separation provides a mechanism for concentrating and segregating cellular components in a spatiotemporally defined manner for a variety of functional processes (Brangwynne, 2013; Hyman et al., 2014).

This opinion paper focuses on the most up-to-date progress of phase separation in regulating autophagy, including autophagic substrates assembly, autophagosome formation, and transcriptional control of autophagy. For a comprehensive summary of developments of phase separation in autophagy, please refer to the recent excellent reviews (Wang and Zhang, 2019; Noda et al., 2020; Sun et al., 2020; Fujioka and Noda, 2021).

### Phase Separation Mediates Autophagy Substrates Assembly

**FIGURE 1:**
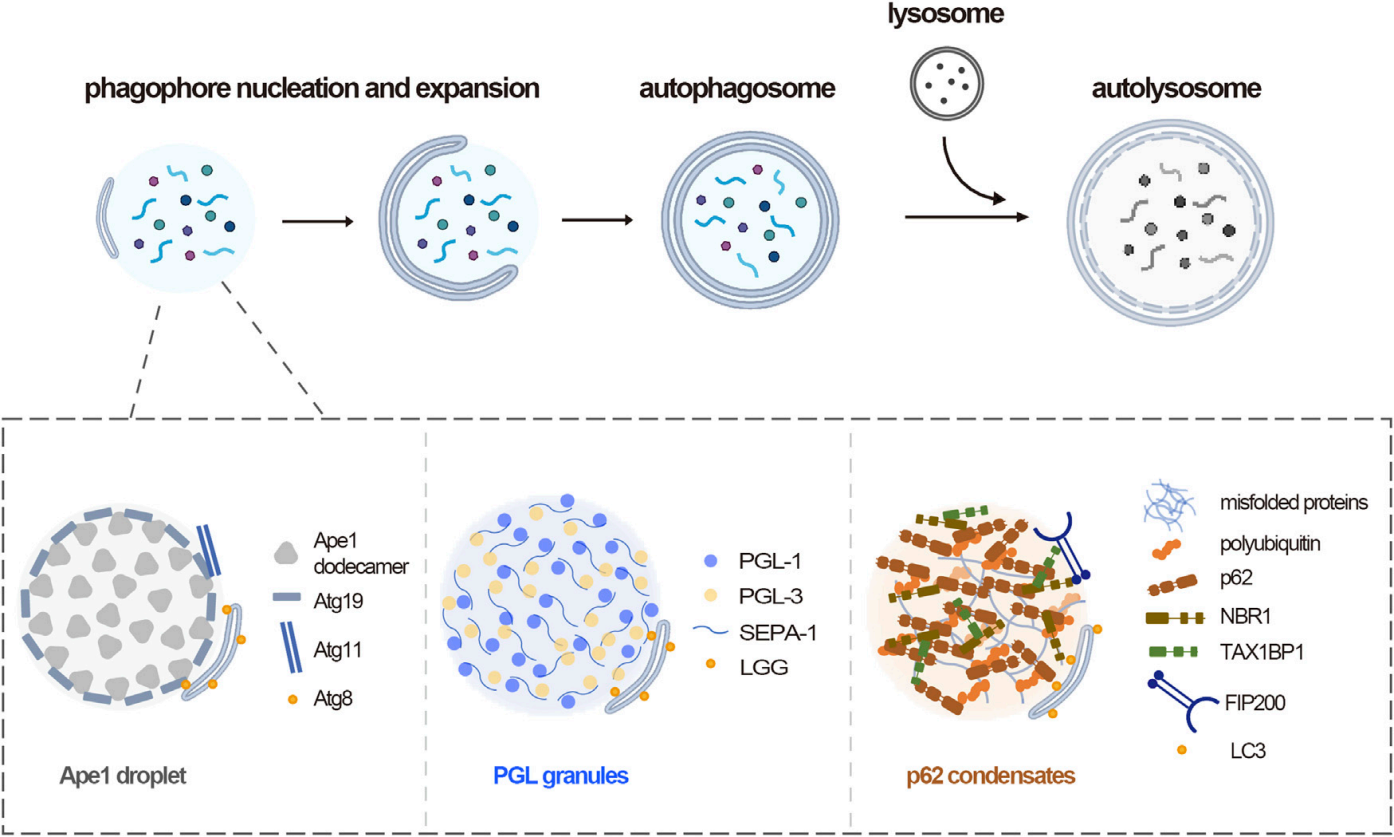
Phase separation mediates autophagy substrates assembly. Upper panel, overview of autophagy process, including phagophore nucleation and expansion, autophagosome formation, fusion between autophagosome and lysosome. Lower panel, three representative autophagy substrates across species assemble through phase separation. In yeast, Ape1 dodecamers form droplets by phase separation, then the receptor Atg19 is recruited, which in turn recruits Atg11. In *C. elegans*, PGL-1 and PGL-3 phase separated into gel-like condensates in somatic cells. In mammals, multivalent interactions between p62 and polyubiquitin drive p62 condensates formation. NBR-1 promotes p62 phase separation and recruits TAX1BP1, which cooperate for the FIP200 recruitment.

So far, the role of phase separation in autophagy is best illustrated in the assembly of selective autophagy substrates. There are two types of autophagy, bulk autophagy and selective autophagy. In bulk autophagy, intracellular materials are non-selectively engulfed by autophagosome. While in selective autophagy, specific cargos including misfolded protein aggregates and damaged organelles are sequestered by autophagosome and a family of cargo receptors are responsible for the specific recognition of different cargos (Kirkin and Rogov, 2019). Accumulated evidences show that multiple selective substrates undergo phase separation for the autophagic degradation across different species (Figure 1).

In budding yeast *S. cerevisiae*, several vacuolar enzymes are constitutively delivered to the vacuole through the selective autophagy-like cytoplasm to vacuole targeting (Cvt) pathway (Lynch-Day and Klionsky, 2010). The aminopeptidase precursor prApe1 self-assembles into a dodecamer and clusters to form Ape1 complex, and is sorted to the vacuole (Klionsky et al., 1992). Indeed, the Ape1 complex was recently identified to be formed by phase separation, which is mediated through weak multivalent interactions between propeptides exposed on the surface of Ape1 dodecamers (Yamasaki et al., 2020). The Ape1 condensates were recognized by the cargo receptor Atg19 which further recruited autophagy machinery Atg11 to trigger autophagosome formation (Kim et al., 2001; Yorimitsu and Klionsky, 2005; Kamber et al., 2015). A Pro-to-Leu mutation at residue 22 of Ape1 that impairing the Cvt pathway inhibited the gel-like condensates formation (Yamasaki et al., 2020). These suggest that the liquid-like property of the Ape1 condensate is important for its degradation by the Cvt pathway.

In *C. elegans*, one well-characterized autophagy substrate that identified to undergo phase separation is the PGL granule. During *C. elegans* embryogenesis, the embryos differentiate to form somatic blastomeres and an immortal germline that is characterized by the presence of the ribonucleoprotein granules, named P granules (Seydoux and Braun, 2006; Strome and Lehmann, 2007). The P granules exhibit liquid-like properties, such as high sphericity and a high propensity to fuse. The granule components, including PGL-1, PGL-3, MEG-3, and LAF-1, were shown to phase separate into droplet *in vitro* (Elbaum-Garfinkle et al., 2015; Saha et al., 2016; Smith et al., 2016). The P granule proteins are originally partitioned into somatic blastomeres but quickly removed by autophagy. PGL-1 and PGL-3 in somatic cells assemble into PGL granule by phase separation (Zhang et al., 2018). The autophagy receptor protein SEPA-1 promotes phase separation of PGL granules (Zhang et al., 2009; Zhang et al., 2018). The scaffold protein EPG-2 determines the size and liquidity of the granules. The methylation of PGL-1 and PGL-3 by EPG-11 inhibits their phase separation. In contrast, the phosphorylation of PGL proteins by mTOR promotes granule formation (Zhang et al., 2018). These data indicate a multilayer regulation of PGL granule formation, which enables the rapid autophagic degradation of PGL proteins in somatic cells.

In mammals, the p62 (also known as SQSTM1) condensates containing polyubiquitinated proteins are a representative of a selective autophagy substrate. p62 was initially identified as a receptor for misfolded protein aggregates (Bjorkoy et al., 2005), however, recent studies show that p62 can form phase-separated condensates with polyubiquitin, depending on its self-oligomerization and binding to ubiquitin (Sun et al., 2018; Zaffagnini et al., 2018). p62 protein alone forms filaments *in vitro*, and undergoes phase separation in the presence of polyubiquitin chains, as the multivalent interactions between p62 and polyubiquitin provide a driving force for the condensate formation. The p62-ubiquitin condensates are low-liquidity droplets, which could serve as a platform to initiate autophagosome biogenesis by recruiting FIP200 (Turco et al., 2019; Kageyama et al., 2021), the scaffold of ULK1 complex. Another receptor NBR1 promotes the formation of p62 condensates and increases their mobility (Sanchez-Martin et al., 2020; Turco et al., 2021). Additionally, NBR1 recruits a third receptor, TAX1BP1 to the p62 condensates and TAX1BP1 in turn drives robust FIP200 recruitment (Turco et al., 2021). In addition, a recent study shows that p62 condensates wet autophagosomal membranes, which could ensure piecemeal or complete condensates sequestration by autophagosomes (Agudo-Canalejo et al., 2021). Other autophagy substrates including the huntingtin protein (Peskett et al., 2018), microtubule-associated protein Tau (Ambadipudi et al., 2017), stress granules (Molliex et al., 2015) were also shown to undergo phase separation.

The intracellular excess or misfolded proteins have long been described to be substrates for autophagy. The identification of these protein substrates that form liquid-like droplets, instead of solid protein aggregates, provides new insights into the regulatory mechanisms for their degradation by autophagy. It reveals a general role of phase separation in the assembly of such protein substrates, which enables the effective autophagic removement. The liquidity property is important for the recognition by autophagosome, which provides new strategies to improve the clearance of disease related protein aggregates. So far, the autophagic cargo receptors was shown to mediate the substrate phase separation, it would be interesting to investigate other autophagy factors or small molecules that regulate the substrate condensate formation or modify the condensate properties. Besides being substrates, these liquid-like proteins droplets could serve as platforms that drive autophagosome formation by recruiting core autophagy machineries and shaping the autophagic membranes, which also needs further investigation.

### Phase Separation in Autophagosome Formation

Recent studies indicate a role of phase separation in autophagosome formation in yeast. The mechanisms of autophagosome formation differs in yeast and mammalian cells. Autophagy is initiated at a particular perivacuolar site called the preautophagosomal structure (PAS) in yeast. The Atg1 complex, consisting of subunits Atg1, Atg13, Atg17, Atg29, and Atg31, is thought to have a central role in recruiting a set of Atg proteins to organize the PAS (Suzuki et al., 2001). A recent study shows that the PAS is actually a liquid-like condensate, and the Atg1 complex could undergo phase separation to form lipid droplet *in vitro* (Fujioka et al., 2020). The IDRs of Atg13 bridges Atg17 dimer (Ragusa et al., 2012; Fujioka et al., 2014; Yamamoto et al., 2016), which drives the phase separation of Atg1 complex *in vitro*. However, given that it takes only tens of Atg13 and Atg17 molecules for phagophore initiation in cells (Geng et al., 2008; Lin et al., 2018), whether phase separation of Atg1 complex occurs at the physiological scale remains to be investigated. The role of phase separation in organizing Atg proteins clustering at the PAS in cells remains to be determined.

A structure that similar to the PAS is not identified in mammalian cells, instead, ATG proteins assemble at multiple sites to initiate autophagosome formation. It is not clear whether these ATG protein assemblies in cells have liquid-like properties. Besides, the phase separation of ULK1 complex, the mammalian counterpart of Atg1 complex, was not observed *in vitro* at nanomolar concentrations (Shi et al., 2020), although ULK1, FIP200, and ATG13 subunits of the ULK1 complex all contain large IDRs (Mei et al., 2014). Whether higher concentration of individual components or posttranslational modifications that affect the kinase activity would promote the phase separation of the whole ULK1 complex both *in vitro* and *in vivo* remains to be determined. And whether phase separation plays a role in autophagosome formation in mammalian cells is largely unknown.

Our recent *in vitro* reconstitution work suggests that instead of phase separation which requires a high protein concentration reaches the critical threshold, a handful autophagy molecules could assemble through a network of multiple low affinity interactions among different components. Such examples include weak interactions between WIPI2 and PI3KC3-C1, WIPI2 and PI(3)P form a positive feedback (Fracchiolla et al., 2020). A higher order assembly of NDP52 via poly-ubiquitin triggers the membrane binding of ULK1 complex (Shi et al., 2020). And a multiplicity of weak interactions between core complexes and cargo receptors drive the ATG8 protein lipidation reaction forward (Chang et al., 2021b). These suggest a multivalent weak interaction web formed by multiple autophagy machineries with low copy numbers could drive the autophagosome formation, which needs further investigation in mammalian cells.

### Phase Separation in the Transcriptional Control of Autophagy

Phase separation is a key mechanism for gene transcriptional control. Both general and signaling pathway specific transcriptional factors undergo phase separation to regulate gene expression (Hnisz et al., 2017; Lu et al., 2020). Similar to other transcriptional factors or coactivators, TFEB, which is responsible for autophagy and lysosome biogenesis gene transcription (Raben and Puertollano, 2016), was also identified to form transcriptional condensates by phase separation (Chen et al., 2020). The inositol polyphosphate multikinase (IPMK) directly interacts with and inhibits TFEB phase separation (Chen et al., 2020). TFEB condensates exhibit low fusion propensity, high interfacial tension and rigid interfacial boundaries (Wang et al., 2022). A high throughput screening of small molecules shows that potent compounds that enhance lysosome function could modify the material properties of TFEB droplets (Wang et al., 2022). As the transcriptional control of autophagy genes is an important mechanism to integrate diverse signaling pathways that regulates autophagy, these reveal a new regulatory mechanism of autophagy and lysosome biogenesis. It would provide a potential therapeutic strategy for lysosomal disorders by investigating new factors that regulate TFEB condensate formation.

### Future Perspective

The findings outlined above highlight the role of phase separation in different stages of autophagy. Given the fundamental function of phase separation in concentrating and segregating intracellular components, it plays crucial roles in the assembly of multiple selective autophagy substrates, enabling their rapid and effective degradation by autophagy. However, how these autophagy substrate condensates recruit core autophagy machineries besides the initial ULK1/Atg1 complex to drive the *de novo* formation of autophagosomes remains unclear. The mechanisms how these substrate condensates shape the autophagic membrane remains to be resolved. In addition, whether phase separation plays roles in other steps of autophagy remains to be illustrated, and it would also be interesting to investigate the roles of phase separation in other membrane remodeling events. Importantly, quantitative studies that determine the molecular copy numbers of autophagy machineries in cells are in need to describe the function of phase separation on the physiological scale. The phase separation in organization of components that involved in autophagy also provides new strategies to develop potential pro-autophagic therapies.
